# The Rho-activating CNF1 toxin from pathogenic *E. coli*: A risk factor for human cancer development?

**DOI:** 10.1186/1750-9378-3-4

**Published:** 2008-03-12

**Authors:** Sara Travaglione, Alessia Fabbri, Carla Fiorentini

**Affiliations:** 1 Department of Therapeutic Research and Medicines Evaluation, Istituto Superiore di Sanità, viale Regina Elena 299, 00161-Rome, Italy

## Abstract

Nowadays, there is increasing evidence that some pathogenic bacteria can contribute to specific stages of cancer development. The concept that bacterial infection could be involved in carcinogenesis acquired a widespread interest with the discovery that *H. pylori *is able to establish chronic infections in the stomach and that this infection is associated with an increased risk of gastric adenocarcinoma and mucosa associated lymphoid tissue lymphoma. Chronic infections triggered by bacteria can facilitate tumor initiation or progression since, during the course of infection, normal cell functions can come under the control of pathogen factors that directly manipulate the host regulatory pathways and the inflammatory reactions.

Renowned publications have recently corroborated the molecular mechanisms that link bacterial infections, inflammation and cancer, indicating certain strains of *Escherichia coli *as a risk factor for patients with colon cancer. *E. coli *is a normal inhabitant of the human intestine that becomes highly pathogenic following the acquisition of virulence factors, including a protein toxin named cytotoxic necrotizing factor 1 (CNF1). This toxin permanently activates the small GTP-binding proteins belonging to the Rho family, thus promoting a prominent polymerization of the actin cytoskeleton as well as a number of cellular responses, including changes in protein expression and functional modification of the cell physiology. CNF1 is receiving an increasing attention as a putative factor involved in transformation because of its ability to: (i) induce COX2 expression, an immediate-early gene over-expressed in some type of cancers; (ii) induce a long-lasting activation of the transcription factor NF-kB, a largely accepted marker of tumor cells; (iii) protect epithelial cells from apoptosis; (iv) ensue the release of pro-inflammatory cytokines in epithelial and endothelial cells; and (v) promote cellular motility. As cancer may arise through dysfunction of the same regulatory systems, it seems likely that CNF1-producing *E. coli *infections can contribute to tumor development.

This review focuses on the aspects of CNF1 activity linked to cell transformation with the aim of contributing to the identification of a possible carcinogenic agent from the microbial world.

## Introduction

### Bacterial infection and cancer

In the last century, cancer research thoroughly established the role of major carcinogenic agents of different nature, including infectious agents. However, although there is a general agreement that some viruses, such as hepatitis B virus (HBV), Epstein-Barr virus (EBV) and human papilloma virus (HPV) can cause cancer, the involvement of bacteria in carcinogenesis remains controversial. The role of viral infections in tumor onset is widely accepted because of the direct action of single viral genes (oncogenes) that result in cell transformation [1]. By contrast, the molecular mechanism(s) by which bacteria might promote tumorigenesis are still poorly characterized. Hence, one of the main challenges, nowadays, is to define the impact of bacterial infections as a cause of cancer and eventually design strategies for their prevention and control.

Bacterial infections are usually believed to cause acute diseases, but it is now becoming clear that some bacteria can contribute to the establishment of chronic diseases, including cancer [2]. The concept that bacterial infection could be involved in carcinogenesis was first proposed in the late nineteenth and early twentieth centuries, based on the discovery of bacteria at the sites of tumors, although there was no proof that the bacteria were in any way causative [3]. Since then, the putative link between chronic infection and cancer acquired a widespread interest with the discovery that *Helicobacter pylori *is able to establish chronic infections in the stomach and that this infection is associated with an increased risk of gastric adenocarcinoma [4] and mucosa associated lymphoid tissue (MALT) lymphoma [5]. In this context, it is worth noting that *H. pylori *is classified as a class I carcinogenic factor [6]. Other chronic bacterial infections have been linked to human carcinogenesis although the underlying mechanisms remain to be defined (reviewed in [2]). The strongest epidemiological case is for *Salmonella enterica *serovar typhi (*S. typhi*), the agent of typhoid, which can also lead to chronic bacterial carriage in the gallbladder [7-11]. Surveys of typhoid outbreaks have shown that those who become carriers have an increased risk of developing hepatobiliary carcinoma compared with people who have had acute typhoid and have cleared the infection [9].

A recurring theme in the link between bacterial infection and carcinogenesis is that of chronic inflammation, which is often a common feature of persistent infection [2,12]. One of the key molecules that link chronic inflammation and cancer is represented by the NF-kB family of transcription factors [12,13]. In particular, different mouse studies provide strong and direct genetic evidence that the classical, IKK-β dependent NF-kB activation pathway is indeed a crucial mediator of tumor promotion [14-16]. This pathway is triggered by bacterial and viral infections, as well as by pro-inflammatory cytokines, such as TNF-α and IL-1, all of which activate the IKK complex [17]. This complex phosphorilates the NF-kB inhibitors IkBs, thereby targeting them for proteosomal degradation and freeing NF-kB to enter the nucleus and mediate transcription of target genes. It is worth noting that many of the genes able to mediate alterations characterizing a tumor cell are under the transcriptional control of NF-kB (reviewed in [18,19]). For example, the activity and expression of cyclin D1, CDK2 kinase, c-myc, p21, p53 and pRb, which are involved in the control of cell cycle and are altered in several types of cancer, are NF-kB-dependent. The expression of numerous cytokines, that are growth factors for tumor cells (IL-1β, TNF, IL-6, EGF) are also regulated by NF-kB. Tissue invasion and metastasis, two crucial events of tumor progression, are regulated by NF-kB-dependent genes, including metalloproteases (MMPs), urokinase type of plasminogen activator (uPA), IL-8, the adhesion molecules VCAM-1, ICAM-1 and ELAM-1. NF-kB is also involved in the regulation of angiogenesis, the process by which tumor cells promote neo-vascularization. Finally, altered expression of genes involved in suppression of apoptosis (i.e. Bcl-2 family members and IAP proteins), a key feature of cancer cells, is often due to deregulated NF-kB activity.

Concerning this last point, several pathogenic bacteria, particularly those that can establish a persistent intracellular infection, activate NF-kB in the host cell and suppress cell death, thus creating a niche in which the bacterium can survive, in spite of the attempts of the host immune system to destroy the infected cell [20]. As a consequence, the suppression of apoptosis by a pathogen might also allow a partially transformed cell to evade the self-destructive process and to progress to a higher level of transformation.

Another important feature of inflammation-associated cancer is the production of reactive oxygen species (ROS) and nitric oxide (NO) by inflammatory and epithelial cells. This leads to increased mutations and altered functions of important enzymes and proteins in inflamed tissue, thus contributing to the multistage carcinogenetic process [21]. For example, during a chronic *H. pylori *infection, production of ROS and nitroxides and the associated inflammatory response are assumed to contribute to the induction of a gastric carcinogenic process [22,23]. These chemical species are mainly produced by inflammatory cells to fight infection and are source of oxidative DNA damage, thus contributing to carcinogenesis [22,23]. In particular, it has been demonstrated that within 6 hours, *H. pylori *infection had a mutagenic effect on gastric epithelial cells [24]. The major form of oxidative DNA damage is the formation of 8-oxoG lesions, specifically repaired by the OGG1 DNA glycosylase. The inactivation of this enzyme inhibits the level of inflammatory lesions and abolishes the mutagenic effect induced by the infection at the gastric level, thus strengthening a close relation between chronic inflammation and genotoxicity [24].

### Bacterial toxins and cancer

As stated above, there is increasing evidence that some pathogenic bacteria can contribute to specific stages of cancer development. In particular, chronic infections triggered by bacteria can facilitate tumor initiation or progression since, during the course of infection, normal cell functions can undergo the control of factors released by the pathogen [2]. These bacterial factors, namely virulence factors, can directly manipulate the host regulatory pathways and the inflammatory reaction [3].

Bacteria express a wide range of virulence factors, including protein toxins that have evolved to interact with eukaryotic cellular machinery in a precise way. These toxins interfere with key eukaryotic processes, such as cellular signaling components, and some directly attack the genome [25-27]. These last can damage DNA via different mechanisms: i) directly by enzymatic attack, ii) indirectly by provoking an inflammatory reaction that produces free radicals, or even iii) by affecting DNA repair mechanisms. Nougayrède and colleagues [28] have recently identified a novel hybrid peptide-polyketide compound from *Escherichia coli *that leads to DNA damage. This novel compound is produced by pathogenic and, most interestingly, commensal isolates. Although it is not yet clear how the peptide-polyketide compound functions at the molecular level, it is possible that it contributes to bacterial pathogenesis and bacterially-induced carcinogenesis.

Any bacterial product that interferes with signaling, resulting in the disruption of the normal balance of growth, cell division and apoptosis, could facilitate tumor promotion. Similarly, the ability to promote anchorage-independent growth could favor metastatic potential and lead to cancer progression. So far, the best example of potentially carcinogenic toxin is *H. pylori *CagA that interferes with cellular signaling mechanisms in a way that is characteristic of tumor promoters. Indeed, CagA intracellularly interacts, in a phosphorylation-dependent and independent way, with many host proteins that regulate cell growth, motility and polarity, thus leading to gastric epithelial proliferation, cell-cell dissociation and increased cell scattering and motility (for a review [29]). In particular, Bagnoli and coworkers [30] showed that CagA is sufficient to disrupt the mechanisms that maintain normal epithelial differentiation, including cell adhesion, cell polarity, and the inhibition of migration. Since the cellular behavior induced by CagA is reminiscent of oncogenes that disrupt cytoskeletal signaling, the authors proposed that altered cell-cell and cell matrix interactions may serve as an early event in *H. pylori*-induced carcinogenesis [30]. Very recently, in experimental studies dealing with *H. pylori *strains carrying or not CagA, it has been demonstrated that this factor activates host cell survival and anti-apoptotic pathways to overcome self-renewal of the gastric epithelium, thus enhancing bacterial colonization of the stomach and helping sustained *H. pylori *infection. In this context, it is interesting to note that, patients infected with *H. pylori *encoding the *cag *pathogeniticy island (PAI) are associated with an increased risk of gastric cancer [31].

In addition to the link between *H. pylori *and stomach cancers, very recent studies evidenced that some other toxins may contribute to bowel and urogenital tract cancers [3]. In this context, it has been reported that adherent and invasive strains of *Escherichia coli *are a risk factor for patients with pre-cancerous and cancerous colon diseases [32,33]. Interestingly, in one of these studies, 3 out of 8 cancer-associated *E. coli *were reported to possess the cytotoxic necrotizing factor 1 (*cnf1*) gene [32], the gene coding for the protein toxin CNF1. The aim of this review is to highlight the main cell responses to CNF1, particularly those related to signaling pathways linked to inflammation and to cell transformation.

## CNF1 from *E. coli*: a protein toxin that activates the Rho GTPases

**Figure 1:**
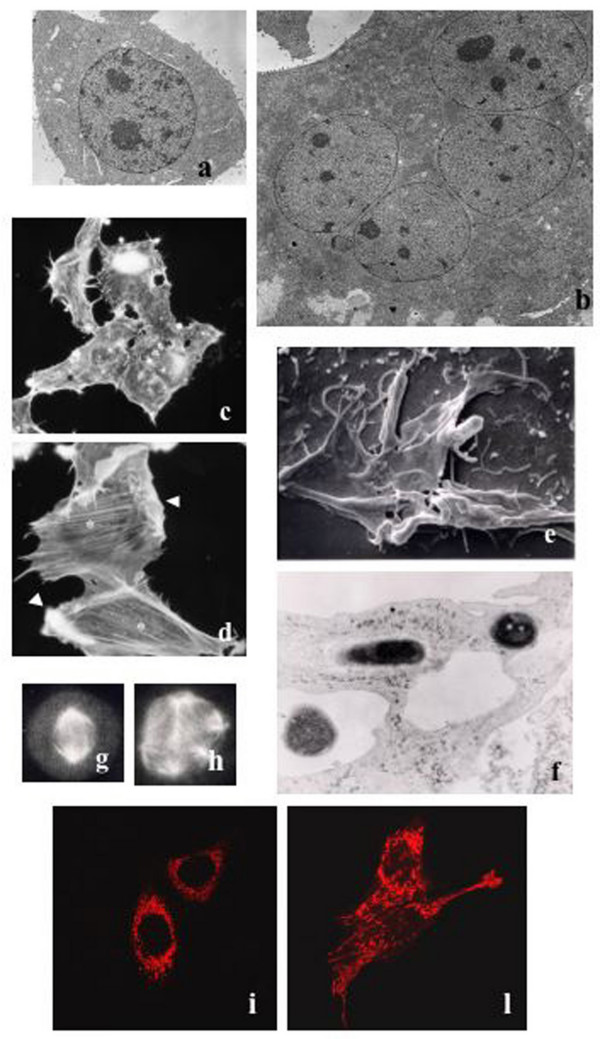
**Different aspects of CNF1 activity on epithelial cells**. **(a-b) **Transmission electron micrographs showing: a control mononucleated cell (a), and a CNF1-treated cell (b), bearing four nuclei in the cytoplasm. **(c-d) **Fluorescence micrographs of control (c) or CNF1-treated (d) cells stained for F-actin detection. CNF1-treated cells display polymerization of actin into stress fibers (asterisks) and prominent ruffles (arrows). **(e-f) **Scanning (e) and transmission (f) electron micrographs showing different stages of the internalization process of non-invasive bacteria by CNF1-treated cells. After being contacted by membrane ruffles (e), bacteria are internalized within vacuoles (f). **(g-h) **Fluorescence micrographs of cells stained with an antibody that recognizes tubulin, the main component of microtubules. Note in h, an example of multipolar mitosis induced by CNF1. **(i-l) **Fluoresce micrographs of control (i) and CNF1-treated (l) cells transfected with the Ds-Red plasmid to visualize mitochondrial organization. CNF1 induces the formation of elongated and interconnected mitochondria.

Although belonging to the normal human intestinal flora, *E. coli *becomes highly pathogenic following the acquisition of genes coding for virulence factors, one of which being CNF1 [34]. CNF1-producing *E. coli *strains are occasionally detected in isolates from feces of children with diarrhea [35-37], but, more frequently, are responsible of extraintestinal infections, particularly in the urinary tract (UTIs) [38-40]. Also, these strains can be detected in cases of bacteraemia [41] and of meningitis in neonates [42]. CNF1, first described in 1983 by Caprioli and coworkers as a toxin capable of causing multinucleation ("cytotoxic") in cultured cells (Fig. 1a, b) and necrosis in rabbit skin ("necrotizing") [43,44], is a single-chain multidomain protein toxin, containing a binding domain to a cell receptor (N-terminal domain), a translocation domain (middle domain) and an enzymatic one (C-terminal domain) [45,46], which modifies a specific cellular target in the host cell cytosol. CNF1 binds to the surface of cultured epithelial cells with high affinity [47], and the 67-kDa laminin receptor has been suggested (67LR) as a putative receptor for CNF1 [48]. After binding to its receptor, CNF1 is endocytosed and routed to an endosomal compartment [47], from where the toxin injects its catalytic activity into the cytosol [47], by using two hydrophobic structures present in the middle part of the CNF1 molecule [49].

The cytoplasmic target of CNF1 is represented by the Rho GTPases, important molecular switches belonging to the Ras superfamily, that cycle between an inactive GDP-bound state and an active GTP-bound one, under the strict control of activators (guanine nucleotide exchange factors, GEFs) and inactivators (GTPase-activating proteins, GAPs) [50]. The conformational changes, induced by the binding of GTP or GDP, occur inside two molecular domains of the Rho proteins called *switches *that are responsible of the coupling of the G-proteins with their downstream effectors (*switch 1*) and allow the interaction of the activated GTPase with GAPs (*switch 2*). The enzymatic activity of CNF1 consists in the deamidation of a specific glutamine residue located in the *switch 2 *domain of the G proteins (glutamine 63 of Rho [51,52] or glutamine 61 of Rac and Cdc42 [53]). This glutamine residue is essential for the GTPase activity of Rho proteins, either intrinsic or GAP-mediated [54]. By modifying glutamine into glutamic acid, CNF1 impairs the role of Rho GAP, allowing Rho proteins to be permanently locked in their activated GTP-bound state and, thus, enhancing the activity of these proteins on their effectors.

It is worth noting that the modification on Rho proteins induced by CNF1 corresponds to the modification found on Ras proteins in many tumors [55]. In fact, Rho glutamine 63 corresponds to Ras glutamine 61, a well-known residue that, after mutagenization, leads to a permanent activation of the molecule and to tumor onset.

## CNF1 and Rho GTPases: actin remodeling and host cell invasion

The Rho GTPases are pivotal in controlling the actin cytoskeleton architecture [50]. In fact, one of the first described cell responses to CNF1 is a remarkable reorganization of the actin cytoskeleton in epithelial cells that consists in the assembly of F-actin in prominent stress fibers, membrane ruffles and filopodia [56] (Fig. 1c, d). The prominent ruffling activity promoted by CNF1 permits epithelial cells to behave as phagocytes, developing a macropinocytotic activity that allows the capture and engulfment of different types of particles, including bacteria [57,58]. This aspect is of particular relevance for the bacterial pathogenicity since the macropinocytic activity ensued by CNF1 in epithelial cells may possibly represent the route of entry of CNF1-producing *E. coli*, similarly to what occurs with other intestinal pathogens (Fig. 1e). Furthermore, upon activation by CNF1, the Rho GTPases undergo sensitization to ubiquitylation and subsequent proteosomal degradation [59], a process that would turn off the ruffling process, thus allowing an efficient internalization of bacteria inside the cells (Fig. 1f). It is worth noting that the activity of CNF1, with its ability to switch on the Rho GTPases and then coerce their degradation in the proteasome, is somehow similar to the activity of the intracellular bacterium *Salmonella *[60], for which a link with cancer has been evidenced [9]. This bacterium first activates the Rho GTPases, by the GEF-like toxin SopE, to promote macropinocytosis that allows its entry into cells and soon after, once inside, deactivates the GTPases *via *a GAP-mimicking protein (SptP), thus allowing a moderate threshold of Rho protein activation for a high invasion efficiency [61].

By regulating the actin cytoskeleton, the Rho GTPases also play a crucial role in certain aspects of the malignant phenotype, such as tumor cell motility, invasiveness and metastasis [62]. Indeed, RhoC has been implicated as a marker for highly angiogenic and aggressive breast cancer with a high metastatic ability [63]. An additional clue in favor of our hypothesis comes from the observation that CNF1 provokes cell junctions disruption and strongly enhances cellular motility in uroepithelial 804G cells [59]. The augmented motility of epithelial cells highlights another characteristic that somehow links this toxin to cancer.

## CNF1 hinders apoptosis via the pro-inflammatory Akt/IKK/NF-kB pathway

It is also known that Rho proteins are crucially involved in the development of inflammatory processes and that a key player in the link existing between Rho, chronic inflammation and cancer is the Nuclear Factor-kB (NF-kB) [64]. As mentioned in the Introduction, NF-kB is represented by a group of structurally related and evolutionarily conserved transcription factors involved in regulating the expression of genes that control different aspects of the tumor cell biology, including inflammation, cell growth, and suppression of apoptosis (reviewed in [65]). Tumor cells can show elevated NF-κB-regulated transcription, which can inhibit TNF-α-induced apoptosis through the up-regulation of the anti-apoptotic proteins of the Bcl-2 family.

In this context, we previously reported that CNF1 can activate the nuclear factor-kB (NF-kB) [66] in epithelial cells. Such an activation, is responsible for the ability of the toxin to stimulate the expression of pro-inflammatory factors and to protect host cell from apoptotic stimuli. As concerns the pro-survival activity, we have shown that CNF1 can increase the expression of proteins related to cell adhesion (integrins, Focal Adhesion Kinase, cadherins, catenins), thus improving cell spreading and the ability of cells to adhere to each other and to the extracellular matrix [67]. In fact, prolonged cell survival, together with increased adhesion to matrix components might have significant biological consequences and affect the tumorigenic potential of epithelial cells. Moreover, CNF1 protects epithelial cells against the drop of the mitochondrial membrane potential provoked by UVB radiation and increases the expression of the anti-apoptotic members of the Bcl-2 family, Bcl-2 and Bcl-X_L _[68]. Although the causal relationship between Bcl-2 and mitochondrial membrane potential has not yet been clarified, we have very recently demonstrated that the up-regulation of the anti-apoptotic protein Bcl-2 somehow controls the mitochondrial morphology, and that this depends on the activation of the pro-inflammatory Rac1/PI3K/Akt/IKK/NF-kB pathway [69]. In fact, besides blocking the activity of the pro-apoptotic members of Bcl-2 family, Bcl-2 is involved in the regulation of mitochondrial morphology, since Bcl-2-over-expressing mitochondria present both increased volume and structural complexity [70]. In living cells, mitochondria continuously divide (fission) and fuse (fusion) with one another [71] and Bcl-2 family members are involved in these processes, the pro-apoptotic members regulating fission whereas the anti-apoptotic regulating fusion of mitochondria [72]. In this context, we very recently demonstrated that CNF1 can induce, in epithelial cells, the formation of a complex network of elongated and interconnected mitochondria with an increased average length [[69]; Fig. 1i, l]. Importantly, Bcl-2 silencing reduces the ability of CNF1 to protect cells against apoptosis and also prevents the CNF1-induced mitochondrial changes. Therefore, since the mitochondrial remodeling is of direct relevance for the role of these organelles in cell physiology and the mitochondrial dysfunction can contribute to a number of human disorders, including cancer [73], the role of CNF1 as a factor favoring transformation can be further supported by this novel finding.

As concerns inflammation, CNF1 ensues the transcription and release of pro-inflammatory cytokines, such as IL-6, IL-8 and TNF-α in uroepithelial [74] and endothelial [75] cells, probably contributing to the establishing of the inflammatory process due to CNF1-producing *E. coli*. The pro-inflammatory role of CNF1 is also supported by the demonstration that the toxin strongly up-regulate the transcription of cyclooxygenase- 2 (COX-2) [76], an immediate-early gene induced in response to pro-inflammatory cytokines, tumor promoters, and growth factors and over-expressed in cancers of the lung, colon, stomach, and breast [77-79].

## Conclusion

**Figure 2:**
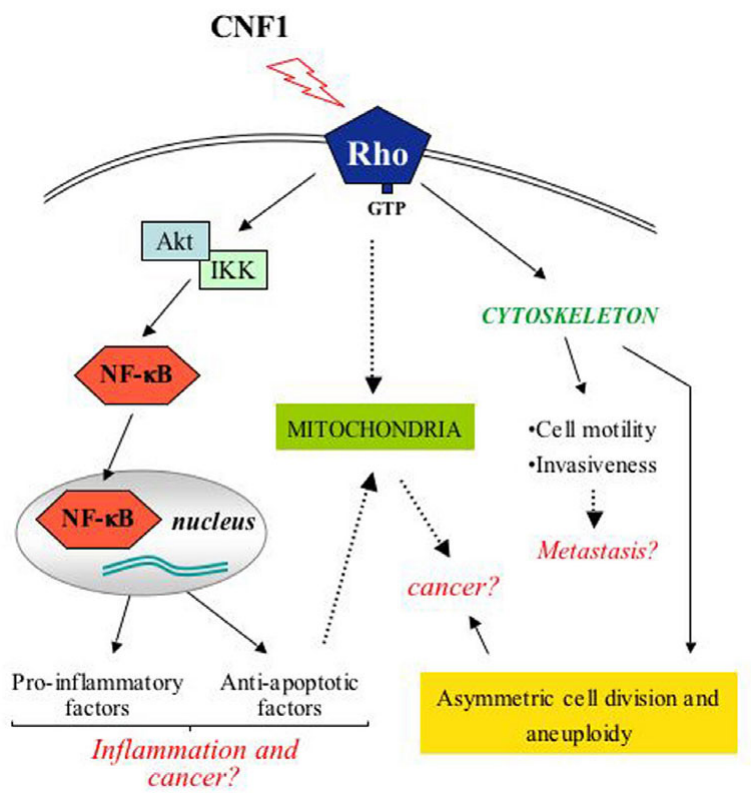
**Hypothetic model on how the pro-inflammatory CNF1 activity can be connected to cancer**. CNF1-dependent Rho activation stimulates NF-κB nuclear translocation and trans-activation, through the classical Akt/IKK-mediated pathway. NF-κB induces the transcription of genes coding for proteins involved in inflammation and apoptosis, that is the pro-inflammatory molecules IL-6, IL-8, TNF-α and Cox-2, and anti-apoptotic factors, such as Bcl-2. This last also causes elongation and enrichment of the mitochondrial network, an aspect somehow linked to transformation. On the other hand, by mean of its activity on the actin cytoskeleton organization, CNF1 is able to provoke cell junctions disruption and to strongly enhance cellular motility, a phenomenon strictly linked to invasiveness and metastasis.

On the whole, it appears that CNF1 touches some of the signaling pathways that are engaged by carcinogens and tumor promoters, as schematized in Fig. 2. Particularly relevant are the activation of the transcription factor NF-κB, the pro-survival activity, the increased expression of RNA messengers for COX-2 and pro-inflammatory cytokines as well as the augmented cell motility. In addition, CNF1 impairs the cytokinesis, thus leading to multinucleation [44,56], induces nuclear segmentation, amitotic cell division, multipolar mitosis (Fig. 1g, h) [80], and modulates autophagy [81], cellular phenomena that are frequently observed in different types of cancer cells, and blocks the cell cycle G2/M transition in epithelial cells [82]. The ability of CNF1 to block the cell cycle progression suggests a strategy that permits to contain the host damage, and to induce specific cellular responses rather than rapid cell death. This could in turn facilitate the bacterial invasion of underlying tissues. The activity of CNF1, with its ability to switch on the Rho GTPases and then coerce their degradation in the proteasome, probably renders the CNF1-producing *E. coli *intracellular parasites, hence permitting their potentially harmful and presumably transforming activity inside the cell, where they can escape the host immune system attack. Finally, it is worth noting that CNF1 activity on cells shares several properties with CagA from the carcinogenic bacterium *H. pylori*, which can be indicated as the best example of a potentially carcinogenic toxin [29]. In Table 1, the cellular effects of *E. coli *CNF1 and those of the major virulence factors (VacA and CagA) of *H. pylori *are compared.

**Table 1:** Comparison between the cellular effects of *E. coli *CNF1 and those of the major virulence factors (VacA and CagA) of *H. pylori*

	**CNF1**	**CagA**	**VacA**
**Effects on cytoskeleton and cell morphology**	• Actin polymerization • Spreading • Multinucleation	• Actin rearrangement • "hummingbird" phenotype • loss of cell polarity • cell scattering	• Actin disruption • Vacuolization
**Apoptosis regulation**	Anti-apoptotic effect	Anti-apoptotic effect	Pro-apoptotic effect
**Production of inflammatory mediators**	TNF-α	IL-8	TNF-α
	IL-6	ROS	IL-8
	IL-8		IL-1β
	ROS		IL-6
**Activation of transcription factors**	NF-kB	NF-kB	NF-kB
		NFAT	ATF-2
		SRF	NFAT
		AP1	
**Effect on cell cycle**	Block in G_2_/M	Inhibition of G_1_/S progression	Induction of G_1_/S progression
**Effects on mitochondria**	Inhibition of UVB-induced mitochondrial membrane depolarization		Reduction of mitochondrial membrane potential, cytochrome c release

Hence, taking altogether, we can hypothesize that once secreted by epithelium-associated *E. coli*, CNF1 switches on the Rho GTPases, initiating a pathway that leads to the actin cytoskeleton reorganization, but also to the activation of NF-κB, which in turn enter the nucleus and causes the transcription of anti apoptotic factors as well as pro-inflammatory cytokines. Production and secretion of pro-inflammatory factors recruits cells of the immune system creating an inflammatory environment. Epithelial cells with the Rho GTPases modified by CNF1 in a way that corresponds to the modification found on Ras proteins in many tumors [55], continue to grow without undergoing apoptosis in an environment rich of inflammatory factors. This can account, finally, for promotion or increase of the neoplastic risk.

## Authors' contributions

ST, AF and CF participated in drafting the manuscript and in its approval.

## References

[B1] Kuper H, Adami H-O, Trichopoulos D (2000). Infections as a major preventable cause of human cancer. J Int Med.

[B2] Lax AJ, Thomas W (2002). How bacteria could cause cancer: one step at a time. Trends Microbiol.

[B3] Lax AJ (2005). Opinion: Bacterial toxins and cancer – a case to answer?. Nat Rev Microbiol.

[B4] Parsonnet J, Friedman GD, Vandersteen DP, Chang Y, Vogelman JH, Orentreich N, Sibley RK (1991). *Helicobacter pylori *infection and the risk of gastric carcinoma. N Eng J Med.

[B5] Wotherspoon AC, Ortiz-Hidalgo C, Falzon MR, Isaacson PG (1991). *Helicobacter pylori*-associated gastritis and primary B-cell gastric lymphoma. Lancet.

[B6] Blaser MJ, Atherton JC (2004). *Helicobacter pylori *persistence: biology and disease. J Clin Invest.

[B7] Welton JC, Marr JS, Friedman SM (1979). Association between hepatobiliary cancer and typhoid carrier state. Lancet.

[B8] Caygill CPJ (1994). Cancer mortality in chronic typhoid and paratyphoid carriers. Lancet.

[B9] Caygill CPJ, Braddick M, Hill MJ, Knowles RL, Sharp JCM (1995). The association between typhoid carriage, typhoid infection and subsequent cancer at a number of sites. Eur J Cancer Prev.

[B10] Nath G, Singh H, Shukla VK (1997). Chronic typhoid carraige and carcinoma of the gladdbladder. Eur J Cancer Prev.

[B11] Shukla VK, Singh H, Pandey M, Upadhyay SK, Nath G (2000). Carcinoma of the gladdbladder-is a sequel of typhoid?. Dig Dis Sci.

[B12] Karin M, Greten FR (2005). NF-κB: linking inflammation and immunity to cancer development and progression. Nature Rev.

[B13] Karin M, Cao Y, Greten FR, Li ZW (2002). NF-κB in cancer: from innocent bystander to major culprit. Nature Rev Cancer.

[B14] Greten FR, Eckmann L, Greten TF, Park JM, Li ZW, Egan LJ, Kagnoff MF, Karin M (2004). IKKbeta links inflammation and tumorigenesis in a mouse model of colitis-associated cancer. Cell.

[B15] Pikarsky E, Porat RM, Stein I, Abramovitch R, Sharon A, Kasem S, Gutkovich-Pyest E, Uriel-Shoval S, Galun E, Ben-Nerlah Y (2004). NF-κB functions as a tumor promoter in inflammation-associated cancer. Nature.

[B16] Luo JL, Maeda S, Hsu LC, Yagita H, Karin M (2004). Inhibition of NF-κB in cancer cells converts inflammation-induced tumor growth mediated by TNF-α to TRAIL-mediated tumor regression. Cancer Cell.

[B17] Häcker H, Karin M (2006). Regulation and function of IKK and IKK-related kinases. Sci STKE.

[B18] Pacifico F, Leonardi A (2006). NF-κB in solid tumors. Biochem Pharmacol.

[B19] Karin M (2006). NF-κB and cancer: mechanisms and targets. Mol Carcinogenesis.

[B20] Van Antwerp DJ, Martin SJ, Kafri T, Green DR, Verma IM (1996). Suppression of TNF-α induced apoptosis by NF-κB. Science.

[B21] Coussens LM, Werb Z (2002). Inflammation and cancer. Nature.

[B22] Farinati F, Cardin R, Degan P, Rugge M, Di Mario F, Bonvicini P, Naccarato R (1998). Oxidative DNA damage accumulation in gastric carcinogenesis. Gut.

[B23] Obst B, Wagner S, Sewing K, Beil W (2000). *Helicobacter pylori *causes DNA damage in gastric epithelial cells. Carcinogenesis.

[B24] Touati E, Michel V, Thiberge J-M, Avé P, Huerre M, Bourgade F, Klungland A, Labigne A (2006). Deficiency in OGG1 protects against inflammation and mutagenic effects associated with H. pylori infection in mouse. Helicobacter.

[B25] Schiavo G, van der Goot FG (2001). The bacterial toxin toolkit. Nat Rev Mol Cell Biol.

[B26] Fiorentini C, Falzano L, Travaglione S, Fabbri A (2003). Hijacking Rho GTPases by protein toxins and apoptosis: molecular strategies of pathogenic bacteria. Cell Death Differ.

[B27] Lax AJ (2007). New genotoxin shows diversity of bacterial attack mechanisms. Trends Mol Med.

[B28] Nougayrède J-P, Homburg S, Taieb F, Boury M, Brzuszkiewicz E, Gottschalk G, Buchrieser C, Hacker J, Dobrindt U, Oswald E *Escherichia coli *induces DNA double-strand breaks in eukaryotic cells. Science.

[B29] Hatakeyama M, Higashi H (2005). *Helicobacter pylori *CagA: a new paradigm for bacterial carcinogenesis. Cancer Sci.

[B30] Bagnoli F, Buti L, Tompkins L, Covacci A, Amieva MR (2005). *Helicobacter pylori *CagA induces a transition from polarized to invasive phenotypes in MDCK cells. Proc Natl Acad Sci USA.

[B31] Blaser MJ, Perez-Perez GI, Kleanthous H, Cover TL, Peek RM, Chyou PH, Stemmermann GN, Nomura A (1995). Infection with *Helicobacter pylori *strains possessing *cagA *is associated with an increased risk of developing adenocarcinoma of the stomach. Cancer Res.

[B32] Martin HM, Campbell BJ, Hart CA, Mpofu C, Nayar M, Singh R, Englyst H, Williams HF, Rhodes JM (2004). Enhanced *Escherichia coli *adherence and invasion in Crohn's disease and colon cancer. Gastroenterology.

[B33] Darfeuille-Michaud A, Boudeau J, Bulois P, Neut C, Glasser AL, Barnich N, Bringer MA, Swidsinski A, Beaugerie L, Colombel JF (2004). High prevalence of adherent-invasive *Escherichia coli *associated with ileal mucosa in Crohn's disease. Gastroenterology.

[B34] Boquet P, Fiorentini C, Aktories K, Just I (2000). The cytotoxic necrotizing factor 1 from *Escherichia coli*. Bacterial Protein toxins Handbook of Experimental Pharmacology.

[B35] Elliott SJ, Srinivas S, Albert MJ, Alam K, Robins-Browne RM, Gunzburg ST, Mee BJ, Chang BJ (1998). Characterization of the roles of hemolysin and other toxins in enteropathy caused by alpha-hemolytic *Escherichia coli *linked to human diarrhea. Infect Immun.

[B36] Okeke IN, Lamikanra A, Steinruck H, Kaper JB (2000). Characterization of *Escherichia coli *strains from cases of childhood diarrhea in provincial southwestern Nigeria. J Clin Microbiol.

[B37] Paciorek J (2002). Virulence properties of *Escherichia coli *faecal strains isolated in Poland from healthy children and strains belonging to serogroups O18, O26, O44, O86, O126 and O127 isolated from children with diarrhoea. J Med Microbiol.

[B38] De Rycke J, Milon A, Oswald E (1999). Necrotoxic *Escherichia coli *(NTEC): two emerging categories of human and animal pathogens. Vet Res.

[B39] Landraud L, Gauthier M, Fosse T, Boquet P (2000). Frequency of *Escherichia coli *strains producing the cytotoxic necrotizing factor (CNF1) in nosocomial urinary tract infections. Lett Appl Microbiol.

[B40] Boquet P (2001). The cytotoxic necrotizing factor 1 (CNF1) from *Escherichia coli*. Toxicon.

[B41] Blanco J, Blanco M, Gonzalez EA, Alonso MP, Garabal JI (1990). Comparative evaluation of three tests for the detection of *Escherichia coli *cytotoxic necrotizing factors (CNF1 and CNF2) using filtrates of cultures treated with mitomycin C. FEMS Microbiol Lett.

[B42] Houdouin V, Bonacorsi S, Brahimi N, Clermont O, Nassif X, Bingen E (2002). A uropathogenicity island contributes to the pathogenicity of *Escherichia coli *strains that cause neonatal meningitis. Infect Immun.

[B43] Caprioli A, Falbo V, Roda LG, Ruggeri FM, Zona C (1983). Partial purification and characterization of an *Escherichia coli *toxic factor that induces morphological cell alterations. Infect Immun.

[B44] Caprioli A, Donelli G, Falbo V, Possenti R, Roda LG, Roscetti G, Ruggeri FM (1984). A cell division-active protein from *E. coli*. Biochem Biophys Res Commun.

[B45] Lemichez E, Flatau G, Bruzzone M, Boquet P, Gauthier M (1997). Molecular localization of the *Escherichia coli *cytotoxic necrotizing factor CNF1 cell-binding and catalytic domains. Mol Microbiol.

[B46] Fabbri A, Gauthier M, Boquet P (1999). The 5' region of cnf1 harbours a translational regulatory mechanism for CNF1 synthesis and encodes the cell-binding domain of the toxin. Mol Microbiol.

[B47] Contamin S, Galmiche A, Doye A, Flatau G, Benmerah A, Boquet P (2000). The p21 Rho-activating toxin cytotoxic necrotizing factor 1 is endocytosed by a clathrin-independent mechanism and enters the cytosol by an acidic-dependent membrane translocation step. Mol Biol Cell.

[B48] Kim KJ, Chung JW, Kim KS (2005). 67-kDa laminin receptor promotes internalization of cytotoxic necrotizing factor 1-expressing *Escherichia coli *K1 into human brain microvascular endothelial cells. J Biol Chem.

[B49] Pei S, Doye A, Boquet P (2001). Mutation of specific acidic residues of the CNF1 T domain into lysine alters cell membrane translocation of the toxin. Mol Microbiol.

[B50] Etienne-Manneville S, Hall A (2002). Rho GTPases in cell biology. Nature.

[B51] Flatau G, Lemichez E, Gauthier M, Chardin P, Paris S, Fiorentini C, Boquet P (1997). Toxin-induced activation of the G protein p21 Rho by deamidation of glutamine. J Biol Chem.

[B52] Schmidt G, Sher P, Wilm M, Selzer J, Mann M, Aktories K (1997). Gln 63 of Rho is deamidated by *Escherichia coli *cytotoxic necrotizing factor-1. Nature.

[B53] Lerm M, Selzer J, Hoffmeyer A, Rapp UR, Aktories K, Schmidt G (1999). Deamidation of Cdc42 and Rac by *Escherichia coli *cytotoxic necrotizing factor 1: activation of the C-Jun N-terminal kinase in HeLa cells. Infect Immun.

[B54] Rittinger K, Walker PA, Eccleston JF, Nurmahomed K, Owen D, Laue E, Gamblin SJ, Smerdon SJ (1997). Crystal structure of a small G protein in complex with the GTPase-activating protein rhoGAP. Nature.

[B55] Bos JL (1989). Ras oncogenes in human cancer: a review. Cancer Res.

[B56] Fiorentini C, Arancia G, Caprioli A, Falbo V, Ruggeri FM, Donelli G (1988). Cytoskeletal changes induced in HEp-2 cells by the cytotoxic necrotizing factor of *Escherichia coli*. Toxicon.

[B57] Falzano L, Fiorentini C, Donelli G, Michel E, Kocks C, Cossart P, Cabanié L, Oswald E, Boquet P (1993). Induction of phagocytic behaviour in human epithelial cells by *Escherichia coli *cytotoxic necrotizing factor type 1. Mol Microbiol.

[B58] Fiorentini C, Falzano L, Fabbri A, Stringaro A, Logozzi M, Travaglione S, Contamin S, Arancia G, Malorni W, Fais S (2001). Activation of rho GTPases by cytotoxic necrotizing factor 1 induces macropinocytosis and scavenging activity in epithelial cells. Mol Biol Cell.

[B59] Doye A, Mettouchi A, Bossis G, Clement R, Buisson-Touati C, Flatau G, Gagnoux L, Piechaczyk M, Boquet P, Lemichez E (2002). CNF1 exploits the ubiquitin-proteasome machinery to restrict Rho GTPase activation for bacterial host cell invasion. Cell.

[B60] Fu Y, Galàn JE (1999). A *Salmonella *protein antagonizes Rac1 and Cdc42 to mediate host-cell recovery after bacterial invasion. Nature.

[B61] Schlumberger MC, Hardt WD (2005). Triggered phagocytosis by *Salmonella *: bacterial molecular mimicry of RhoGTPase activation/deactivation. Curr Top Microbiol Immunol.

[B62] Ridley AJ (2004). Rho proteins and cancer. Breast Cancer res Treat.

[B63] Kleer CG, van Golen KL, Zhang Y, Wu ZF, Rubin MA, Merajver SD (2002). Characterization of RhoC expression in benign and malignant breast disease. Am J Pathol.

[B64] Marx J (2004). Inflammation and cancer: the link grows stronger. Cancer Res.

[B65] Ghosh S, May MJ, Kopp EB (1998). NF-kappa B and Rel proteins: evolutionarily conserved mediators of immune responses. Annu Rev Immunol.

[B66] Boyer L, Travaglione S, Falzano L, Gauthier NC, Popoff MR, Lemichez E, Fiorentini C, Fabbri A (2004). Rac GTPase instructs Nuclear Factor-kB activating by conveying the SCF complex and IkB-alpha to the ruffling membranes. Mol Biol Cell.

[B67] Fiorentini C, Matarrese P, Straface E, Falzano L, Donelli G, Boquet P, Malorni W (1998). Rho-dependent cell spreading activated by *E. coli *cytotoxic necrotizing factor 1 hinders apoptosis in epithelial cells. Cell Death Differ.

[B68] Fiorentini C, Matarrese P, Straface E, Falzano L, Fabbri A, Donelli G, Cossarizza A, Boquet P, Malorni W (1998). Toxin-induced activation of Rho GTP-binding protein increases Bcl-2 expression and influences mitochondrial homeostasis. Exp Cell Res.

[B69] Giamboi Miraglia A, Travaglione S, Meschini S, Falzano L, Matarrese P, Quaranta MG, Viora M, Fiorentini C, Fabbri A (2007). Cytotoxic necrotizing factor 1 prevents apoptosis via the Akt/IkappaB kinase pathway: role of nuclear factor-kappaB and Bcl-2. Mol Biol Cell.

[B70] Kowaltowski AJ, Cosso RG, Campos CB, Fiskum G (2002). Effect of bcl-2 overexpression on mitochondrial structure and function. J Biol Chem.

[B71] Cereghetti GM, Scorrano L (2006). The many shape of mitochondrial death. Oncogene.

[B72] Delivani P, Adrain C, Taylor RC, Duriez PJ, Martin SJ (2006). Role for CED-9 and Egl-1 as regulators of mitochondrial fission and fusion dynamics. Mol Cell.

[B73] Alirol E, Martinou JC (2006). Mitochondria and cancer: is there a morphological connection?. Oncogene.

[B74] Falzano L, Quaranta MG, Travaglione S, Filippini P, Fabbri A, Viora M, Donelli G, Fiorentini C (2003). Cytotoxic necrotizing factor 1 enhances reactive oxygen species-dependent transcription and secretion of proinflammatory cytokines in human uroepithelial cells. Infect Immun.

[B75] Munro P, Flatau G, Doye A, Boyer L, Oregioni O, Mege JL, Landraud L, Lemichez E (2004). Activation and proteasomal degradation of Rho GTPases by cytotoxic necrotizing factor-1 elicit a controlled inflammatory response. J Biol Chem.

[B76] Thomas W, Ascott ZK, Harmey D, Slice LW, Rozengurt E, Lax AJ (2001). Cytotoxic necrotizing factor from *Escherichia coli *induces RhoA-dependent expression of the cyclooxygenase-2 gene. Infect Immun.

[B77] Mann JR, Dubois RN (2004). Cyclooxygenase-2 and gastrointestinal cancer. Cancer J.

[B78] Wang D, Dubois RN (2004). Cyclooxygenase-2: a potential target in breast cancer. Semin Oncol.

[B79] Ristimaki A (2004). Cyclooxygenase 2: from inflammation to carcinogenesis. Novartis Found Symp.

[B80] Malorni W, Fiorentini C (2006). Is the Rac GTPase-activating toxin CNF1 a smart hijacker of host cell fate?. FASEB J.

[B81] Fiorentini C, Malorni W (2006). Exploiting cell death pathways by an E. coli cytotoxin: autophagy as a double-edged sword for the host. Autophagy.

[B82] Falzano L, Filippini P, Travaglione S, Miraglia AG, Fabbri A, Fiorentini C (2006). *Escherichia coli *cytotoxic necrotizing factor 1 blocks cell cycle G2/M transition in uroepithelial cells. Infect Immun.

